# Circulating sex-steroids and *Staphylococcus aureus* nasal carriage in a general female population

**DOI:** 10.1530/EJE-20-0877

**Published:** 2020-12-16

**Authors:** Dina B Stensen, Lars Småbrekke, Karina Olsen, Guri Grimnes, Christopher Sivert Nielsen, Johanna U E Sollid, Gunnar Skov Simonsen, Bjørg Almås, Anne-Sofie Furberg

**Affiliations:** 1 Department of Community Medicine, Faculty of Health Sciences, UiT The Arctic University of Norway, Tromsø, Norway; 2 Division of Internal Medicine, University Hospital of North Norway, Tromsø, Norway; 3 Department of Pharmacy, Faculty of Health Sciences, UiT The Arctic University of Norway, Tromsø, Norway; 4 Division of Internal Medicine, Department of Microbiology and Infection Control, University Hospital of North Norway, Tromsø, Norway; 5 Endocrinology Research Group, Department of Clinical Medicine, Faculty of Health Sciences, UiT The Arctic University of Norway, Tromsø, Norway; 6 Division of Chronic Diseases and Ageing, Norwegian Institute of Public Health, Oslo, Norway; 7 Division of Emergencies and Critical Care, Department of Pain Management and Research, Oslo University Hospital, Oslo, Norway; 8 Research Group for Host-Microbe Interaction, Department of Medical Biology, Faculty of Health Sciences, UiT The Arctic University of Norway, Tromsø, Norway; 9 Hormone Laboratory, Department of Medical Biochemistry and Pharmacology, Haukeland University Hospital, Bergen, Norway; 10 Faculty of Health and Social Sciences, Molde University College, Molde, Norway

## Abstract

**Objective:**

*Staphylococcus aureus* is a major human pathogen, and nasal carriers have an increased risk for infection and disease. The exploration of host determinants for nasal carriage is relevant to decrease infection burden. Former studies demonstrate lower carriage prevalence in women and among users of progestin-only contraceptives. The aim of this study was to investigate the possible associations between circulating sex-steroid hormones and nasal carriage of *Staphylococcus aureus* in a general population.

**Methods:**

In the population-based sixth Tromsø study (2007–2008) nurses collected nasal swab samples from 724 women aged 30–87 not using any exogenous hormones, and 700 of the women had a repeated nasal swab taken (median interval 28 days). We analysed a panel of serum sex-steroids by liquid chromatography tandem mass spectrometry, and collected information about lifestyle, health and anthropometric measures. Multivariable logistic regression was used to study the association between circulating sex-steroids and *Staphylococcus aureus* carriage (one swab) and persistent carriage (two swabs), while adjusting for potential confounding factors. Women in luteal phase were excluded in the analysis of androgens.

**Results:**

*Staphylococcus aureus* persistent nasal carriage prevalence was 22%. One standard deviation increase in testosterone and bioavailable testosterone was associated with lower odds of persistent nasal carriage, (OR = 0.57; 95% CI = 0.35–0.92 and OR = 0.52, 95% CI = 0.30–0.92) respectively. Analysis stratified by menopause gave similar findings. Persistent carriers had lower average levels of androstenedione and DHEA, however, not statistically significant.

**Conclusion:**

This large population-based study supports that women with lower levels of circulating testosterone may have increased probability of *Staphylococcus aureus* persistent carriage.

## Introduction

*Staphylococcus aureus* is the leading cause of skin and soft tissue infections, and can invade and infect any organ of the human body. *S. aureus* is the second most common cause of bloodstream infections (1), which continue to have high mortality among patients worldwide (2, 3). The burden of disease and mortality reflect *S. aureus’* extraordinary virulence factors, including highly evolved immune evasion strategies and resistance to antibiotic treatment (4).

*S. aureus* is frequent in the normal bacterial flora of healthy humans, and 20–50% of the population are nasal carriers (4, 5, 6, 7). Colonisation of the skin in the anterior nares is a major source of endogenous *S. aureus* infections and transmission (4). This motivates the search for lifestyle and environmental factors and associated biomarkers that may regulate immune responses to *S. aureus*. Among populations from different continents, male sex, younger age, atopic eczema, excess body weight, higher circulating glucose levels and diabetes, lower vitamin D levels, and work in health care services have been associated with higher probability of *S. aureus* nasal carriage, while smoking has been associated with both lower and higher probability of carriage (4, 5, 8, 9, 10, 11, 12, 13).

Sex-steroids are produced from cholesterol in testes or ovaries and the adrenal glands, and may be further converted to more potent sex-steroids in fat and other tissue. Sex-differences in immunological responses are largely caused by sex-steroid hormone actions, through binding to nuclear receptors and regulation of immune system genes expression in a variety of cells (14). In general, adult women have a more responsive immune system with faster clearance of pathogens and greater vaccine efficacy compared to men but are more prone to inflammatory and autoimmune diseases. Sex and age are the strongest risk factors for *S. aureus* nasal carriage (4, 8, 9). Thus, it has been hypothesised that sex-steroid hormones are important in the immune response to *S. aureus*. Smaller studies among women have found a positive association of nasal *S. aureus* carriage with biomarkers of endogenous oestrogen level (15), as well as of nasal *S. aureus* carriage with exogenous oestrogen and progesterone (i.e. hormonal contraceptive combination preparations) (16, 17). One small study among premenopausal women found higher prevalence of persistent throat carriage of *S. aureus* with increasing levels of free fraction testosterone (18). From the population-based sixth Tromsø study (2007–2008), we reported higher nasal carriage among men and sex-differences in the distribution of *S. aureus* spa types (genetic variance by *S. aureus* protein A (spa) typing) (9). Among 400 young women in the Tromsø study fit futures (2012–2013), where 50% used hormonal contraception, we found a positive dose-response relationship between oestrogen dose and *S. aureus* nasal carriage, and a negative association between exogenous progesterone alone and nasal carriage (17). To our knowledge, no epidemiological study has examined whether endogenous sex-hormone levels are associated with *S. aureus* nasal carriage among women. Thus, the aim of this study is to investigate the possible associations between circulating sex-steroid hormones and nasal carriage of *S. aureus* in a general female population.

## Methods

### Population and study design

The Tromsø study includes seven extensive health-screening surveys in the Tromsø municipality, North Norway, during 1974–2016 with invitation of total birth cohorts and large random samples of the population (5, 11). Each of these surveys consists of clinical examinations including anthropometric measures, non-fasting blood samples, interview covering menstrual history and hormonal medication, questionnaires on reproductive history, lifestyle, health and chronic disease (5, 11, 12). The present study uses data from the sixth Tromsø study (the sixth Tromsø study, participation proportion 66%).

Body height in centimetres (cm) and weight in kilograms (kg) were measured to the nearest 0.1 unit with participants wearing light clothing and no shoes. BMI was calculated as weight divided by height squared (kg/m^2^). Non-fasting blood samples were drawn from an antecubital vein. Glycated haemoglobin (HbA1c) was determined in EDTA-blood by HPLC using an automated analyser (Variant II, Bio-Rad Laboratories Inc.). The total analytical coefficient of variation was <3.0%. Serum 25-hydroxyvitamin D (25(OH)D) was analysed by electrochemiluminescence immunoassay (ECLIA) using an automated clinical chemistry analyser (Modular E170, Roche Diagnostics). The total analytical coefficient of variation was 7.3%. There is a known overestimation of 25(OH)D levels in smokers when using the ECLIA (Roche) method (19). This necessitates stratification by smoking in the statistical analysis of 25(OH)D.

**Figure 1:**
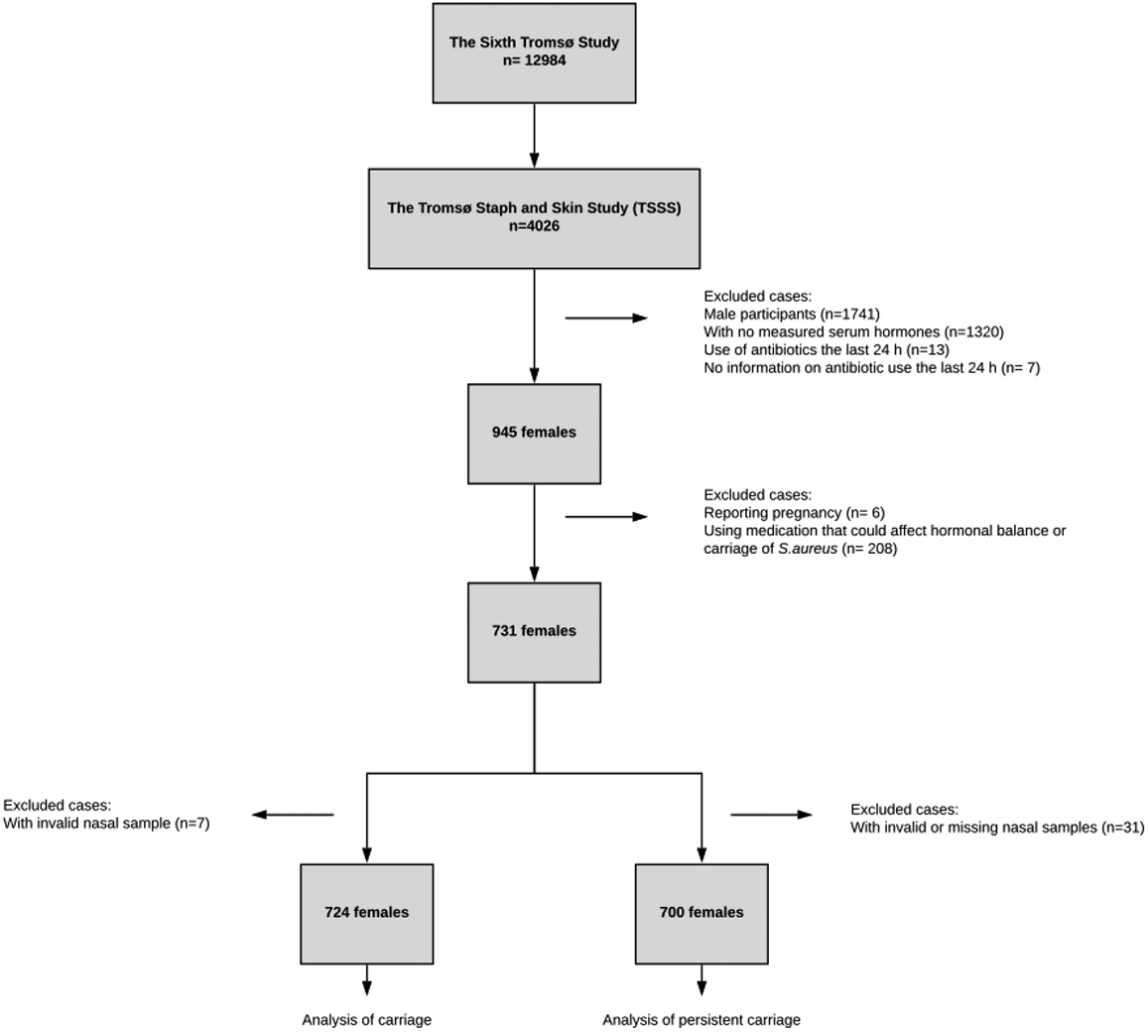
The study population. The sixth Tromsø study.

Nasal swabs for *S. aureus* culturing were collected from a random sample of 4026 participants attending the sixth Tromsø study screening centre during October 2007 to June 2008, 2285 of these were women. All women without data on serum hormones, participants reporting antibiotic use or missing data on antibiotic use the last 24 h, ongoing pregnancy, use of hormonal contraceptives, use of hormonal replacement therapy, endocrine breast cancer therapy and IVF treatment were excluded from the analysis. The participants were invited to a second visit for repeated nasal swab sampling. Women with invalid or missing *S. aureus* culturing results were excluded from the analysis (Fig. 1).

### Assessment of ***S. aureus*** carriage and serum sex-steroids

Nurses at the sixth Tromsø study screening centre collected nasal swab samples. Each nasal vestibule was sampled with the same NaCl (0.9%)-moistened sterile rayon-tipped swab which was rotated three times. The swabs were immediately placed in transport medium (Amies Copan, Brescia, Italy) and stored at 4°C for a maximum of 3 days. Trained personnel at the Department of Microbiology and Infection Control, University Hospital of North Norway, (UNN) Tromsø analysed the microbiological samples. The specimens were cultured on blood agar (Oxoid, UK) for growth control and chromID-plates (SAID) for *S. aureus* detection (bioMérieux, Marcy I’Etoile, France). The agar plates were incubated for 42–48 h at 37°C. To retain high specificity, colony morphology was examined, and the most dominating colony type on the SAID plate was confirmed as *S. aureus* by the Staphaurex plus agglutination test (Murex Diagnostic Ltd, Dartford, UK). Growth of any bacterial colonies on agar plates was registered as a valid culture.

The median interval between the first and the second nasal swab culture was 28 days.

*S. aureus* nasal carriage status was classified according to baseline sample status. *S. aureus* persistent carriage was defined as having two positive samples on the basis of van Belkum’s definition of persistent carriage (20).

The Hormone laboratory at Haukeland University Hospital analysed a panel of serum sex-steroids by liquid chromatography tandem mass spectrometry (LCMS/MS, SCIEX API 5500 triple-quadrupole mass spectrometer, Applied Biosystems/MDS with an Agilent 1290 UPLC system), as part of the laboratory’s work on establishing reference values within subgroups of the general population (i.e. by sex, age, and BMI categories) (21). LCMS/MS is the gold standard method for steroid profiling due to very high sensitivity and specificity (22). Serum concentrations of testosterone, androstenedione, dehydroandrostenedione (DHEAS), 17α-hydroxyprogesterone (17-OH progesterone), progesterone, luteinising hormone (LH), follicle-stimulating hormone (FSH), as well as the binding proteins sex-hormone binding globulin (SHBG) and albumin were measured using DPC immulite 200 XPi (Siemens Healthcare Diagnostics). For the estimation of bioavailable testosterone which includes free and albumin-bound testosterone we used the equation ‘(testosterone/SHBG) × 10’ (23) and the equation derived by Morris *et al.* (24).

### Classification of menopause and menstrual phase

**Figure 2:**
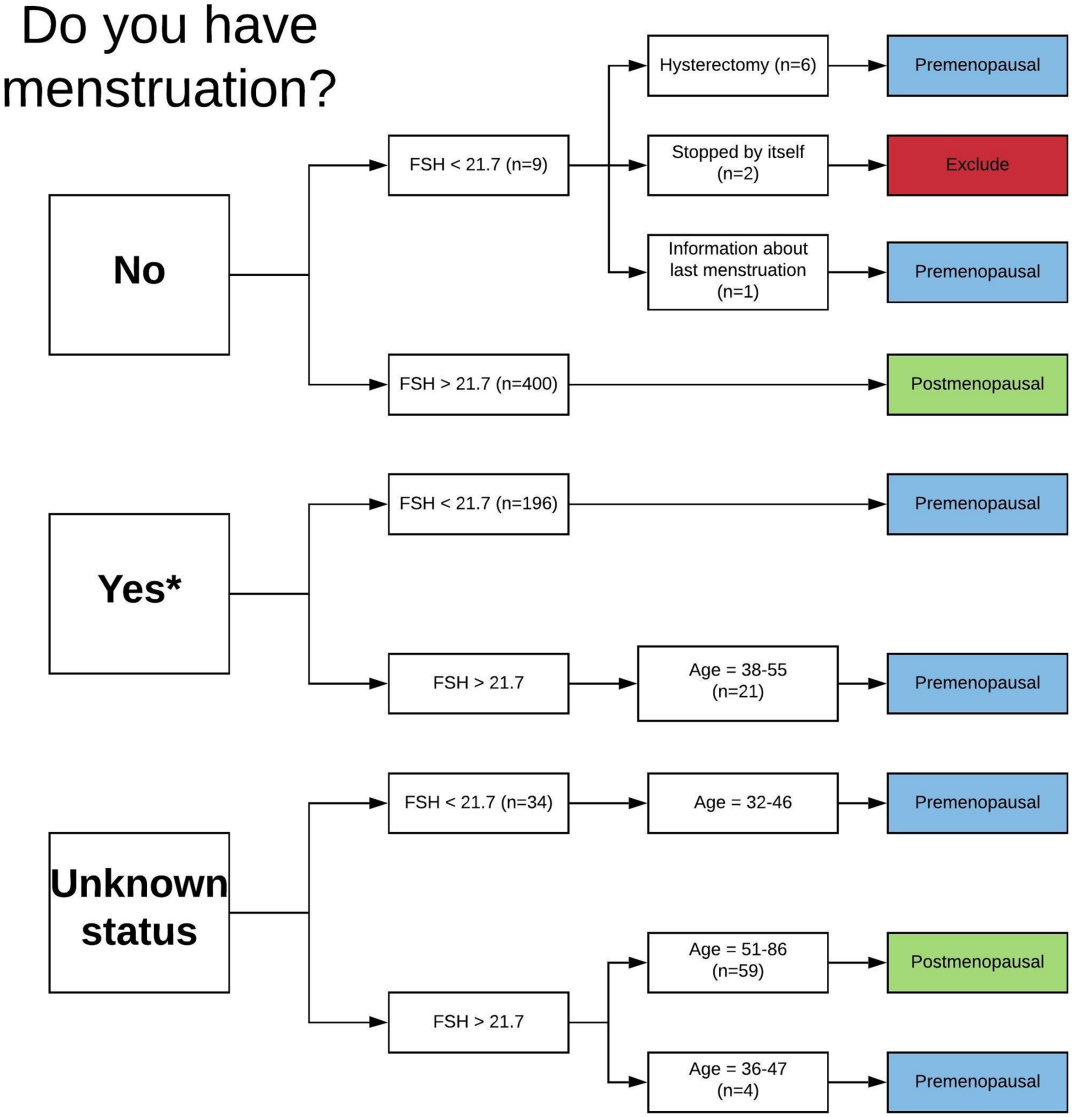
Classification of menstruation status, the sixth Tromsø study. ‘Do you have menstruation?’ corresponds with the interview question ‘Do you still have natural menstruation?’ *Participants reporting irregular menstruation (*n* = 2) are reclassified as ‘yes’. FSH in IU, age in years. A full colour version of this figure is available at https://doi.org/10.1530/EJE-20-0877.

To classify menopausal status, we used the following question from the interview: ‘Do you still have natural menstruation?‘ (yes/no/irregular/unknown status). We also defined FSH-level over 21.7 IU as postmenopausal and FSH-level under 21.7 IU as premenopausal (25, 26). Female participants reporting no menses with FSH-levels under 21.7 IU were further classified by the question from the interview: ‘If you do not have natural menstruation, why did it stop?‘ (stopped by itself/I had a hysterectomy/both ovaries were removed/other reasons (for instance radiation, cytotoxic mediation)). FSH-levels can be difficult to interpret because of the possible high levels in perimenopause, we therefore emphasised self-reported menstruation in classification of menopause. FSH-levels were used to confirm clinical history and to classify females with no data on self-reported menses. Among women with unknown status on menstruation from the interview, age was used as an additional criterium in those with FSH-levels above 21.7 IU (age ≥ 51 years were classified as postmenopausal). One female participant reported no menses but had information about recent menstruation, we therefore concluded that this probably were due to misreporting on menstruation and classified the participant by FSH levels (Fig. 2).

Premenopausal women were further classified as follicular phase or luteal phase according to progesterone levels. Follicular phase were defined as progesterone levels ≤5.5 nmol/L and luteal phase as progesterone levels >5.5 nmol/L (27). In the analysis including premenopausal women, women in the luteal phase are excluded to increase accuracy of the testosterone measurement (28).

### Statistical analysis

Statistical analysis was performed using Stata/MP 15.1 for Macintosh. Univariable associations were analysed in contingency tables by calculating means and s.d. using chi-square and t-test to quantify the potential role of chance. Independent samples t-test were used to test possible associations between sex-steroid hormones, carriage and persistent carriage. Multivariable logistic regression was fitted to estimate odds ratios (ORs) and 95% CIs to describe the associations between sex-steroid hormones and *S. aureus* nasal carriage and *S. aureus* persistent nasal carriage while adjusting for potential confounders. Missing values were excluded from the logistic regression analysis. DAGitty 3.0 was used for model selection (29). Testing for potential interaction between explanatory variables was done by including the multiplicative terms of two predictor variables in the model. *P*-values of ≤0.05 were considered statistically significant.

### Ethics

Each participant in the sixth Tromsø study signed a declaration of consent. The study does not include data from participants with their declaration of consent withdrawn after participation. Tromsø 6 was approved by the Regional Committee for Medical and Health Research Ethics (REK) and the Norwegian Data Protection Authority. The present analysis was approved by REK (2018/1975/REK nord).

## Results

In this population of female participants with age 30–87 years (mean = **53.5, s.d . = 13.6), the prevalence of *S. aureus* nasal carriage was 24% (180/724), and of *S. aureus* persistent nasal carriage 22% (151/700) (Table 1 and Supplementary Table 1, see section on supplementary materials given at the end of this article).

**Table 1:** Characteristics of the study population by *Staphylococcus aureus* nasal carrier state. The sixth Tromsø study. Data are presented as mean ± S.D. or as *n* (%).

	Carrier state, *n* = 724			Persistent carrier state, *n* = 700		
	Non-carrier	Carrier	*P*-value^a^	Others^b^	Persistent carrier	*P*-value^a^
*n*	544	180		549	151	
Age, years	55.9 ± 13.4	55.1 ± 13.7	0.485	56.1 ± 13.3	56.7 ± 13.3	0.628
BMI, kg/m^2^	27.6 ± 5.2	28.2 ± 5.6	0.204	27.5 ± 5.1	28.4 ± 5.8	0.069
Menstruation phase						
Menopause	346 (63.6 %)	113 (62.8 %)	0.935	356 (64.9 %)	103 (68.2 %)	0.487
Luteal phase	87 (16.0 %)	28 (15.5 %)		87 (15.8 %)	18 (11.9 %)	
Follicular phase	111 (20.4 %)	39 (21.7 %)		106 (19.3 %)	30 (19.9 %)	

*Chi-square test for categorical and t-tests for continuous variables; ^b^others; Intermittent carriers (one positive nasal samples of two samples in total) *n* = 49; non-carriers (two negative nasal samples of two samples in total) *n* = 500.

In postmenopausal women, mean level of testosterone were significantly lower in persistent nasal carriers compared to others (mean difference (MD) = 0.10 95% CI = 0.00–0.20) (Table 2). When postmenopausal women’s carrier state was assessed from only one nasal swab, the mean level of bioavailable testosterone was 0.15 nmol/L for non-carriers and 0.13 nmol/L for carriers (MD = 0.02 95% CI = −0.00 to 0.04) (Supplementary Table 2). In postmenopausal women, serum levels of androstenedione, DHEAS, and 17α-hydroxyprogesterone were lower in nasal carriers than in others but not statistically significant (Table 2).

**Table 2:** Mean difference and CI in circulating sex-steroids, gonadotropins and binding protein levels among *Staphylococcus aureus* persistent nasal carriers compared to others. The sixth Tromsø study.

	Premenopausal, *n* = 237^a,b^		Postmenopausal, *n* = 445^a^	
	Mean difference^c^	95% CI^d^	Mean difference^c^	95% CI^d^
Testosterone, nmol/L	0.22	−0.38 to 0.83	0.10	0.00–0.20
Bioavailable testosterone^e^, nmol/L	0.04	−0.11 to 0.18	0.18	−0.00 to 0.04
Androstenedione, nmol/L	0.17	−0.42 to 0.76	0.12	−0.13 to 0.36
Dehydroepiandrosterone, nmol/L	−0.04	−0.93 to 0.85	0.33	−0.04 to 0.69
17α-hydroxyprogesterone, nmol/L	0.88	−2.44 to 4.20	0.14	−0.18 to 0.46
Progesterone, nmol/L	1.57	−3.54 to 6.69	0.04	−0.04 to 0.11
Sex-hormone binding globulin, nmol/L	9.91	0.73–19.09	−0.89	−7.72 to 5.93
Albumin, nmol/L	0.05	−0.83 to 0.93	0.18	−0.42 to 0.78
Luteinising hormone, IU	−0.26	−2.99 to 2.47	0.80	−1.67 to 3.26
Follicle-stimulating hormone, IU	2.68	−1.65 to 7.01	1.39	−4.43 to 7.21

^a^Number may vary due to missing values; ^b^women in luteal phase are excluded in the analysis of testosterone, bioavailable testosterone, androstenedione and DHEA; ^c^mean difference = mean (others) – mean (persistent carriage); ^d^independent sample t-test; ^e^bioavailable testosterone calculated from the equation ‘(testosterone/SHBG) × 10’.

Using DAGitty (29), age and BMI were identified as sufficient covariates in the regression analysis when estimating the association between sex-steroids and *S. aureus* nasal carriage in women. In a multivariable logistic regression model adjusted for age and BMI, an increase of 1 s.d. (s.d. = 0.81 nmol/L) of testosterone lowered the odds of nasal carriage of *S. aureus* (OR = 0.60; 95% CI = 0.39–0.94) as well as persistent nasal carriage (OR = 0.57; 95% CI = 0.35–0.92; s.d . = 0.82 nmol/L) (Table 3).

**Table 3:** Associations between testosterone, bioavailable testosterone and *Staphylococcus aureus* nasal carriage and persistent carriage. Analysis on menopausal women and premenopausal women in follicular phase. Odds ratios (ORs) and 95% CIs from logistic regression analysis. The sixth Tromsø study.

	Nasal carriage	Persistent nasal carriage
Testosterone		
*n*	573	560
per s.d.^a^	0.60 (0.39–0.94)	0.57 (0.35–0.92)
BMI, kg/m^2^	1.03 (0.99–1.07)	1.03 (0.99–1.07)
Age, year	0.99 (0.98–1.01)	1.00 (0.98–1.02)
Bioavailable testosterone		
*n*	557	544
per s.d.^b,c^	0.53 (0.32–0.90)	0.52 (0.30–0.92)
BMI, kg/m^2^	1.05 (1.01–1.09)	1.06 (1.01–1.10)
Age, year	0.99 (0.98–1.01)	1.00 (0.98–1.01)

^a^Serum testosterone (nmol/L) s.d.: analysis of nasal carriage, s.d. = 0.81; analysis of persistent nasal carriage, s.d. = 0.82; ^b^bioavailable testosterone (nmol/L) s.d.: analysis of nasal carriage, s.d. = 0.19; analysis of persistent nasal carriage, s.d. = 0.19; ^c^ioavailable testosterone calculated from the equation: (testosterone/SHBG) × 10.

When stratifying by menopausal status, this decrease in odds of persistent nasal carriage by higher testosterone was also found in postmenopausal women (OR = 0.74; 95% CI = 0.55–0.99; s.d. = 0.43 nmol/L). In premenopausal women, the association was similar but not statistically significant (OR = 0.35; 95% CI = 0.05–2.29; s.d. = 1.48 nmol/L). The odds of nasal carriage by higher testosterone were lower but not statistically significant for premenopausal women (OR = 0.30; 95% CI = 0.06–1.56; s.d. = 1.41 nmol/L) and for postmenopausal women (OR = 0.78; 95% CI = 0.59–1.02; s.d. = 0.43 nmol/L).

Multivariable logistic regression analysis showed a lower odds of nasal carriage (OR = 0.53; 95% CI = 0.32–0.90) and persistent nasal carriage (OR = 0.52; 95% CI = 0.30–0.92) for an increase in bioavailable testosterone by one standard deviation (s.d. = 0.19 nmol/L) (Table 3).

As for testosterone a decrease in odds of persistent nasal carriage was also found for postmenopausal women (OR = 0.72; 95% CI = 0.52–0.99; s.d. = 0.09 nmol/L). In premenopausal women, there was a similar association (OR = 0.38; 95% CI = 0.06–2.45; s.d. = 0.34 nmol/L). The odds of nasal carriage were lower but not statistically significant for premenopausal women (OR = 0.28; 95% CI = 0.05–1.46; s.d. = 0.34 nmol/L) and for postmenopausal women (OR = 0.75; 95% CI = 0.56–1.01; s.d. = 0.09 nmol/L).

**Figure 3:**
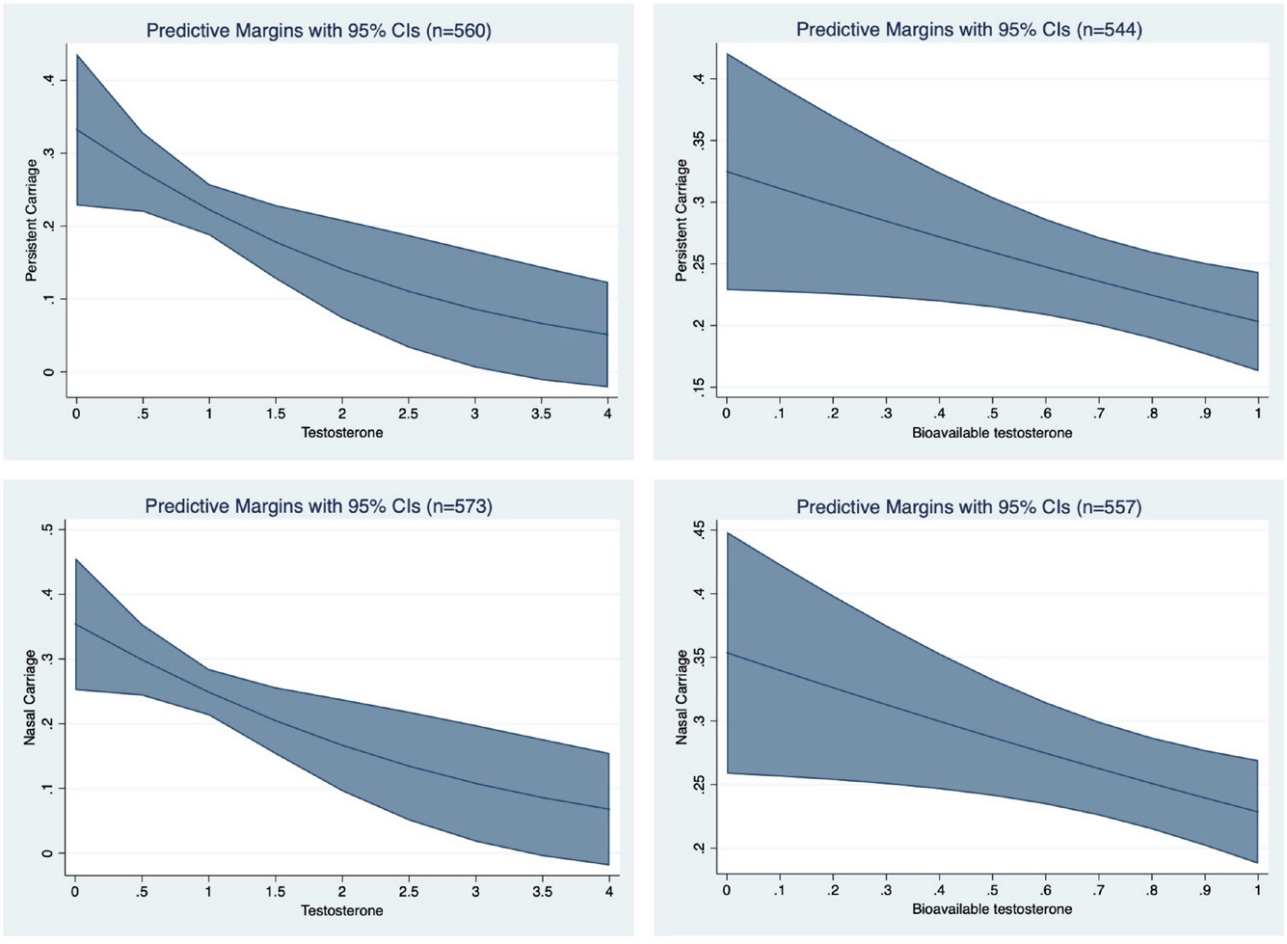
Probability of *S. aureus* nasal carriage (top) and persistent nasal carriage according to testosterone (left) and bioavailable testosterone (right). Analysis of postmenopausal women and premenopausal women in follicular phase. Testosterone and bioavailable testosterone per nmol/L. Bioavailable testosterone calculated from the equation ‘(testosterone/SHBG) × 10‘. A full colour version of this figure is available at https://doi.org/10.1530/EJE-20-0877.

There was an inverse dose-response relationship between bioavailable testosterone and *S. aureus* carriage and persistent carriage, as well as for testosterone. In this female population, 98% had serum testosterone levels between 0 and 2 nmol/L, the estimates for values over 2 nmol/L are therefore more uncertain (Fig. 3).

Interaction analysis between all variables in the final model revealed no significant interactions. When the equation by Morris *et al.* was used to calculate bioavailable testosterone there was no change in the risk estimates for *S. aureus* carriage (Supplementary Table 5) (24). One female participant had an extreme value of testosterone (17.42 nmol/L, population-mean: 0.8 nmol/L). Estimates from the final model did not differ when including or excluding this observation, and the presented results therefore include this observation.

## Discussion

In this large population-based study of women not using any exogenous hormones, we report novel evidence of the association between circulating testosterone and bioavailable testosterone and *S. aureus* nasal carriage in women. This is to our knowledge the first study of circulating sex-steroids and* S. aureus* nasal carriage in a general female population. With increasing testosterone by one standard deviation, we see a 40% decrease in odds of *S. aureus* nasal carriage and a 43% decrease in *S. aureus* persistent nasal carriage compared to non-carriers and others (at least one of two nasal swab cultures negative for *S. aureus*), respectively. The same inverse associations are present in analysis stratified by menopausal status (range of ORs from 0.30 to 0.78), but statistically significant only for persistent nasal carriage in postmenopausal women.

We report a lowering in odds of *S. aureus* nasal carriage and *S. aureus* persistent nasal carriage of 47 and 48%, respectively, with increase of bioavailable testosterone by one standard deviation compared to non-carriers and others. In postmenopausal women, the association with *S. aureus* persistent carriage was weaker, but statistically significant. All other estimates from analysis stratified by menopausal status show the same inverse associations between bioavailable testosterone and both nasal carriage and persistent nasal carriage, range of ORs from 0.28 to 0.75, however, not statistically significant. Possibly, we lack the statistical power to identify a significant association because of the low number of participants in the subpopulations of premenopausal women (*n* = 135–148). Persistent nasal carriers also had lower average levels of androstenedione and DHEAS, however not statistically significant.

The knowledge of host response during *S. aureus* nasal carriage is limited. The presence of *S. aureus* nasal carriage induces both innate and adaptive immune responses, but the bacteria overcome host defence mechanisms to establish carrier status (6, 30). Innate immunity exerts the first response against microbes, and the association between delayed response of toll-like receptor 2 in mice and nasal carriage has been demonstrated (31). In animal models, hypogonadism is associated with an increase in immune response with castration of male mice increasing autoimmune encephalomyelitis severity (32) and the incidence and severity of thyroiditis and adjuvant arthritis in rats (33). A recent study demonstrated an immunomodulating role of dihydrotestosterone in both human and rat vaginal smooth muscle cells (34). In summary, studies in animals document a significant effect of sex-steroid hormones on the immune system, but the results from in vitro studies are conflicting (35). Testosterone has been characterised as immunosuppressive, but recent research suggests that testosterones immunomodulatory effects depend on the reproductive context and menopausal status. There are also inconsistency of results for testosterone in both postmenopausal and premenopausal women where testosterone has been characterised as both pro-inflammatory and anti-inflammatory (36).

Former studies have shown a positive association between combination-hormonal contraceptives and *S. aureus* nasal carriage, but lower odds of carriage with progestin-only contraceptives. Studies have demonstrated lower levels of bioavailable testosterone in women using combined oral contraceptives (37). This supports the finding of lower odds of *S. aureus* nasal carriage in women with higher bioavailable testosterone. Progestin is a synthetic progestogen and is classified from its chemical structure as a derivate of testosterone or progesterone (38). Therefore, progestin has both androgen and progestogen activity when used in contraceptives, and progestin-only users may have a higher prevalence of androgenic side effects than combination-contraceptive users because of the lack of the oestrogen component (39).

High androgen and testosterone levels stimulate the sebaceous glands to increased sebum production, and plays an important role in the development of acne (40). In this study, 50–75% of women with polycystic ovary syndrome have high values of bioavailable testosterone and some of the diagnostic criteria are hyperandrogenistic features as acne (41). These hormonally related changes may also affect the microbiota of the skin and promote bacteria like *Cutibacterium acnes* that suppress the growth of other bacteria like* S. aureus* (42). This may be a plausible biological mechanism contributing to the lower probability of carriage of *S. aureus* with higher testosterone in our study.

Testosterone levels in women, as oestrogen levels, decline in the fourth decade of life and after menopause. Though the biological role of testosterone in women remains largely unclear, androgens are biological precursors of oestrogen production (43). High level of testosterone may therefore represent higher level of estrogen. However, oestrogen was not measured in our study. Estrogen has been theorised to have both pro-inflammatory and anti-inflammatory effects (44). One study found high testosterone and estradiol, and low SHBG levels associated with conditions that represent low-grade inflammation processes (45). The lower odds of *S. aureus* carriage with high levels of testosterone may be an indirect effect of possibly corresponding high levels of oestrogen or the unknown ratio between testosterone and oestrogen.

The major circulating steroids classified as androgens include DHEAS, androstenedione and testosterone (46). The lower levels of DHEAS and androstenedione in *S. aureus* persistent nasal carriers among postmenopausal women support our findings of lower odds of *S. aureus* carriage with higher levels of bioavailable testosterone and testosterone (Supplementary Fig. 2). This may represent an unknown effect of androgens on the immune system and increases the validity of the main finding.

We found no statistically significant association between androstenedione, LH, FSH, DHEAS, 17-OH progesterone, progesterone, SHBG, albumin and *S. aureus* nasal carriage and persistent carriage. This may be due to more complex relationships between sex-steroid hormones and the immune system. For premenopausal women, the female sex-steroid hormones have cyclic variations and data based on one measurement may not be representative for the participants’ hormonal status.

It has been reported that the equation ‘(testosterone/SHBG × 10)‘ is not a reliable when SHBG concentration is low (47). Our analysis does not differ when using either this equation or the equation by Morris *et al.* (24) for the calculation of bioavailable testosterone. This may be due to our study population with mostly healthy women with normal BMI that has a low prevalence of very low measurements of SHBG (48, 49).

We used DAGitty for model selection, and the recommended confounders to adjust for in the analysis were BMI, HbA1c and age (Supplementary Fig. 1). Because of the minor insignificant effect on the main analysis of HbA1c, we decided to only adjust for BMI and age. We report a significant decrease of *S. aureus* with smoking, and a significant association with alcohol use. Because of the decision of not doing model selection by data driven statistical selection process, we did not include these variables in the final logistic regression model (Supplementary Tables 3 and 4). We include tables in Supplementary information for the reader to explore the odds ratio estimates including different possible covariates. The odds ratio estimates changed only slightly in these models. Although our data do not support any interactions among the explanatory variables, we cannot rule out the presence of smaller interaction effects that can only be detected by larger sample sizes.

Strengths of our study include a population-based design with high attendance, and consequently reduced risk of selection bias. Trained nurses collected nasal swab samples, and analyses of blood samples were performed with current gold standard assays for analysing sex-steroid hormones. Using both medical history and circulating FSH-levels to classify premenopausal and menopausal women reduces misclassification bias. One possible bias in the study is misclassification of *S. aureus* carrier status due to only two nasal swab samples, as van Belkum* et al.* recommend seven samples to correctly classify persistent carriers from intermittent carriers. Intermittent carriers has a lower risk of infections similar to non-carriers as well as similar elimination kinetics (20). Thus, we may have an unknown number of intermittent carriers misclassified as persistent in our study, which may have biased the risk estimates towards the null. However, large population-based data reduces the risk of selection bias and increases both the internal and external validity.

In conclusion, we report evidence of an association between circulating testosterone and bioavailable testosterone and *S. aureus* nasal carriage defined from both one and two swab samples in females. Higher levels of bioavailable testosterone are associated with lower odds for *S. aureus* nasal carriage of 39–43%. The role of testosterone and bioavailable testosterone in *S. aureus* nasal carriage ought to be addressed in future prospective studies to identify risk groups for prevention of* S. aureus* carriage and disease.

## Supplementary Material

Supplementary Table 1: Characteristics of the study population by Staphylococcus aureus nasal carrier state; additional covariates included. The 6th Tromsø Study

Supplementary Table 2: Mean difference and confidence intervals (CI) in circulating sex-steroids, gonadotropins and binding protein levels among Staphylococcus aureus nasal carriers compared to others. The 6th Tromsø Study

Supplementary Table 3: Associations between testosterone and Staphylococcus aureus nasal carriage and persistent carriage. Odds ratios (OR) and 95% confidence intervals (95% CI) from logistic regression analysis. The 6th Tromsø Study

Supplementary Table 4: Associations between bioavailable testosteronea and Staphylococcus aureus nasal carriage and persistent nasal carriage.Odds ratios (OR) and 95% confidence intervals (95% CI) from logistic regression analysis. The 6th Tromsø Study

Supplementary Table 5: Associations between bioavailable testosterone and Staphylococcus aureus nasal carrier and persistent carrier state by Morris et ala. Odds ratios (ORs) and 95% confidence intervals (95% CIs) from logistic regression analysis. The Tromsø Study 6

## Declaration of interest

The authors declare that there is no conflict of interest that could be perceived as prejudicing the impartiality of this study.

## Funding

This work was supported by the Northern Norway Regional Health Authority (grant number HNF1457–19) and the Odd Berg Group Medical Research fund 2007.
