# Circulating sex-steroids and *Staphylococcus aureus* nasal carriage in a general female population

**DOI:** 10.1530/EJE-20-0877e

**Published:** 2021-05-13

**Authors:** Dina B Stensen, Lars Småbrekke, Karina Olsen, Guri Grimnes, Christopher Sivert Nielsen, Johanna U E Sollid, Gunnar Skov Simonsen, Bjørg Almås, Anne-Sofie Furberg

**Affiliations:** 1 Department of Community Medicine, Faculty of Health Sciences, UiT The Arctic University of Norway, Tromsø, Norway; 2 Division of Internal Medicine, University Hospital of North Norway, Tromsø, Norway; 3 Department of Pharmacy, Faculty of Health Sciences, UiT The Arctic University of Norway, Tromsø, Norway; 4 Division of Internal Medicine, Department of Microbiology and Infection Control, University Hospital of North Norway, Tromsø, Norway; 5 Endocrinology Research Group, Department of Clinical Medicine, Faculty of Health Sciences, UiT The Arctic University of Norway, Tromsø, Norway; 6 Division of Chronic Diseases and Ageing, Norwegian Institute of Public Health, Oslo, Norway; 7 Division of Emergencies and Critical Care, Department of Pain Management and Research, Oslo University Hospital, Oslo, Norway; 8 Research Group for Host-Microbe Interaction, Department of Medical Biology, Faculty of Health Sciences, UiT The Arctic University of Norway, Tromsø, Norway; 9 Hormone Laboratory, Department of Medical Biochemistry and Pharmacology, Haukeland University Hospital, Bergen, Norway; 10 Faculty of Health and Social Sciences, Molde University College, Molde, Norway

The authors of the above titled article published in the *European Journal of Endocrinology* (vol 184 Iss 2 pages 337–346) apologise for errors in the ‘Methods’ section. The authors state that the instruments for the analysis of testosterone, androstenedione, dehydroandrostenedione, 17α-hydroxyprogesteron and progesterone were incorrectly reported in the ‘Methods’ section: Assessment of *S. aureus* carriage and serum sex-steroids. The correct instruments for the analysis of the given hormones were LCMS/MS, SCIEX API 5500 triple-quadrupole mass spectrometer, Applied Biosystems/MDS with an Agilent 1290 UPLC system. Luteinising hormone, follicle-stimulating hormone, sex-hormone binding globulin and albumin were measured using DPC immulite 200 XPi and not as published.

