# Supplementary material for: Circulating sex-steroids and Staphylococcus aureus nasal carriage in a general female population
Source: Eur J Endocrinol. 2020 Dec 16;184(2):337–46. doi: 10.1530/EJE-20-0877 (PMC7849480; doi:10.1530/EJE-20-0877)
Supplement: Supplementary Table 1: Characteristics of the study population by Staphylococcus aureus nasal carrier state; additional covariates included. The 6th Tromsø Study [file supplementary_table_1.pdf]

Supplementary Table 1: Characteristics of the study population by *Staphylococcus aureus* nasal carrier state; additional covariates included. The 6th Tromsø Study

	Carrier state n=724[a]			Persistent carriage state n=700[a]		
	**Non-carrier** N (%) n= 544	**Carrier** N (%) n=180	**P-value** [b]	**Others**[c] N (%) n= 549	**Persistent carrier** N (%) n=151	**P-value** [b]
**Age, years** (mean, SD)	55.9 (13.4)	55.1 (13.7)	0.485	56.1 (13.3)	56.7 (13.3)	0.628
**BMI, kg/m2** (mean, SD)	27.6 (5.2)	28.2 (5.6)	0.204	27.5 (5.1)	28.4 (5.8)	0.069
**Menstruation phase**						
Menopause	346 (63.6)	113 (62.8)	0.935	356 (64.9)	103 (68.2)	0.487
Luteal phase	87 (16.0)	28 (15.5)		87 (15.8)	18 (11.9)	
Follicular phase	111 (20.4)	39 (21.7)		106 (19.3)	30 (19.9)	
**Smoking**						
Yes	110 (20.9)	22 (12.5)	0.014	112 (21.0)	16 (10.9)	0.005
No	417 (79.1)	132 (87.5)		421 (79.0)	131 (89.1)	
**Alcohol use**						
More than 4 times a month	93 (17.5)	26 (14.9)	0.008	97 (18.0)	19 (13.0)	0.014
2-4 times a month	181 (33.9)	46 (26.3)		179 (33.3)	41 (28.1)	
Once a month or less	182 (34.1)	85 (48.5)		184 (34.2)	71 (48.6)	
Never	77 (14.5)	18 (10.3)		78 (14.5)	15 (10.3)	
**HbA1c, %** [d] (mean, SD)	5.6 (0.6)	5.6 (0.8)	0.555	5.6 (0.6)	5.7 (0.8)	0.168
**Vitamin D nmol/L (non-smokers)**[ef] (mean, SD)	55.4 (18.9)	53.2 (17.1)	0.220	55.1 (18.8)	54.2 (17.5)	0.620
**Vitamin D nmol/L (smokers)**[ef] (mean, SD)	77.6 (21.5)	78.87 (23.7)	0.801	77.5 (21.3)	80.1 (25.8)	0.663
**Hospital admission last 12 months**						
Yes	60 (11.3)	25 (14.4)	0.284	125 (20.9)	20 (24.7)	0.430
No	470 (88.7)	149 (85.6)		474 (79.1)	61 (75.3)	

[a]Number may vary due to missing values
[b]Chi-square test for categorical and t-tests for continuous variables
[c]Others; Intermittent carriers (one positive nasal samples of two samples in total) n=49; Non-carriers (two negative nasal samples of two samples in total) n=500
[d]HbA1c = EDTA-blood glycated hemoglobin (HbA1c)
[e]Vitamin D = serum 25-hydroxyvitamin D [25(OH)D]
[f]Vitamin D is stratified by smoking because of known overestimating of 25(OH)D levels in smokers by unknown mechanisms when using ECLIA
