# Supplementary material for: Circulating sex-steroids and Staphylococcus aureus nasal carriage in a general female population
Source: Eur J Endocrinol. 2020 Dec 16;184(2):337–46. doi: 10.1530/EJE-20-0877 (PMC7849480; doi:10.1530/EJE-20-0877)
Supplement: Supplementary Table 2: Mean difference and confidence intervals (CI) in circulating sex-steroids, gonadotropins and binding protein levels among Staphylococcus aureus nasal carriers compared to others. The 6th Tromsø Study [file supplementary_table_2.pdf]

Supplementary Table 2: Mean difference and confidence intervals (CI) in circulating sex-steroids, gonadotropins and binding protein levels among *Staphylococcus aureus* nasal carriers compared to others. The 6th Tromsø Study

	Premenopausal n=261[ab]		Postmenopausal n=445[a]	
	**Mean difference**[c]	**95 % CI**[d]	**Mean difference**[c]	**95 % CI**[d]
**Testosterone** nmol/L	0.23	-0.29-0.76	0.09	-0.01-0.18
**Bioavailable testosterone**[e] nmol/L	0.04	-0.08-0.17	0.02	-0.00-0.04
**Androstenedione** nmol/L	0.29	-0.26-0.85	0.07	-0.16-0.31
**Dehydroepiandrosterone** nmol/L	0.06	-0.74-0.85	0.13	-0.23-0.48
**17α-hydroxyprogesterone** nmol/L	1.01	-1.76-3.78	0.16	-0.16-0.47
**Progesterone** nmol/L	0.74	-3.68-5.16	0.03	-0.04-0.11
**Sex-hormone binding globulin** nmol/L	5.73	-2.17-13.64	-1.16	-7.76-5.44
**Albumin** nmol/L	0.09	-0.66-0.85	0.23	-0.35-0.81
**Luteinizing hormone** IU	1.12	-1.68-3.91	0.96	-1.43-3.35
**Follicle-stimulating hormone** IU	2.95	-0.66-6.57	0.92	-4.72-6.56

[a]Number may vary due to missing values
[b]Women in luteal phase are excluded in the analysis of testosterone, bioavailable testosterone, androstenedione and dehydroepiandrosterone.
[c]Mean difference = mean (others) – mean (nasal carriage)
[d]Independent sample t-test
[e]Bioavailable testosterone calculated from the equation “(testosterone/SHBG) X 10”
