# Supplementary material for: Circulating sex-steroids and Staphylococcus aureus nasal carriage in a general female population
Source: Eur J Endocrinol. 2020 Dec 16;184(2):337–46. doi: 10.1530/EJE-20-0877 (PMC7849480; doi:10.1530/EJE-20-0877)
Supplement: Supplementary Table 3: Associations between testosterone and Staphylococcus aureus nasal carriage and persistent carriage. Odds ratios (OR) and 95% confidence intervals (95% CI) from logistic regression analysis. The 6th Tromsø Study [file supplementary_table_3.pdf]

Supplementary Table 3: Associations between testosterone and *Staphylococcus aureus* nasal carriage and persistent carriage. Odds ratios (OR) and 95% confidence intervals (95% CI) from logistic regression analysis. The 6th Tromsø Study

	Nasal carriage			Persistent nasal carriage		
	**All women** (n=567)[ab]	**Pre-menopausal** (n=147)[ab]	**Post-menopausal** (n=421)[a]	**All women** (n=554)[ab]	**Pre-menopausal** (n=134)[ab]	**Post-menopausal** (n=421)[a]
**Testosterone**[cd]	0.61 (0.38-0.95)	0.30 (0.06-1.55)	0.79 (0.60-1.04)	0.57 (0.36-0.94)	0.34 (0.05-2.25)	0.75 (0.57-1.00)
**Testosterone**[ce]	0.64 (0.41-1.00)	0.34 (0.06-1.86)	0.81 (0.62-1.06)	0.62 (0.38-1.00)	0.40 (0.06-2.80)	0.78 (0.59-1.04)
**Testosterone**[cf]	0.65 (0.41-1.02)	0.32 (0.06-1.75)	0.83 (0.64-1.08)	0.62 (0.38-1.00)	0.31 (0.04-2.32)	0.80 (0.60-1.06)
**Testosterone**[cg]	0.69 (0.44-1.08)	0.33 (0.06-1.86)	0.86 (0.66-1.11)	0.66 (0.41-1.07)	0.30 (0.04-2.30)	0.83 (0.63-1.09)

	Smokers		Non-Smokers	
	**Carriage**	**Persistent carriage**	**Carriage**	**Persistent carriage**
	**All women**[b] (n=98)	**All women**[b] (n=96)	**All women**[b] (n=428)	**All women**[b] (n=417)
**Testosterone**[ch]	0.53 (0.13-2.05)	0.56 (0.12-2.64)	0.69 (0.43-1.11)	0.64 (0.38-1.07)

[a]Number may vary due to missing values
[b]Women in luteal phase are excluded
[c]Testosterone divided by the standard deviation; Nasal carriage all women SD=0.81; Nasal carriage premenopausal SD=1.41.3387801; Nasal carriage postmenopausal SD=0.43; Persistent carriage all women SD=0.82; Persistent carriage premenopausal SD=1.47; Persistent carriage postmenopausal SD=0.43
[d]Adjusted for BMI, age and HbA1c
[e]Adjusted for BMI, age, HbA1c and smoking
[f]Adjusted for BMI, age, HbA1c, smoking and alcohol use
[g]Adjusted for BMI, age, HbA1c, smoking alcohol use and hospital admission
[h]Adjusted for BMI, age, HbA1c, alcohol use, hospital admission and vitamin D
