# Supplementary material for: Circulating sex-steroids and Staphylococcus aureus nasal carriage in a general female population
Source: Eur J Endocrinol. 2020 Dec 16;184(2):337–46. doi: 10.1530/EJE-20-0877 (PMC7849480; doi:10.1530/EJE-20-0877)
Supplement: Supplementary Table 4: Associations between bioavailable testosteronea and Staphylococcus aureus nasal carriage and persistent nasal carriage.Odds ratios (OR) and 95% confidence intervals (95% CI) from logistic regression analysis. The 6th Tromsø Study [file supplementary_table_4.pdf]

Supplementary Table 4: Associations between bioavailable testosterone[a] and *Staphylococcus aureus* nasal carriage and persistent nasal carriage. Odds ratios (OR) and 95% confidence intervals (95% CI) from logistic regression analysis. The 6th Tromsø Study

	Nasal carriage			Persistent nasal carriage		
	**All women** (n=551)[bc]	**Pre-menopausal** (n=147)[bc]	**Post-menopausal** (n=405)[b]	**All women** (n=538)[bc]	**Pre-menopausal** (n=134)[bc]	**Post-menopausal** (n=405)[b]
**Bioavailable testosterone**[de]	0.53 (0.31-0.90)	0.27 (0.05-1.39)	0.76 (0.56-1.02)	0.52 (0.30-0.91)	0.36 (0.06-2.28)	0.72 (0.53-0.99)
**Bioavailable testosterone**[df]	0.54 (0.31-0.91)	0.25 (0.04-1.36)	0.77 (0.57-1.04)	0.53 (0.30-0.93)	0.32 (0.05-2.22)	0.73 (0.53-1.01)
**Bioavailable testosterone**[dg]	0.54 (0.32-0.91)	0.25 (0.05-1.33)	0.77 (0.57-1.04)	0.52 (0.29-0.92)	0.28 (0.04-1.91)	0.74 (0.53-1.02)
**Bioavailable testosterone**dh	0.56 (0.32-0.94)	0.30 (0.05-1.61)	0.78 (0.58-1.06)	0.54 (0.30-0.95)	0.31 (0.05-2.15)	0.75 (0.54-1.04)

	Smokers		Non-Smokers	
	**Carriage**	**Persistent carriage**	**Carriage**	**Persistent carriage**
	**All women**[c] (n=94)	**All women**[c] (n=92)	**All women**[c] (n=416)	**All women**[c] (n=405)
**Bioavailable testosterone**[di]	0.86 (0.40-1.81)	0.97 (0.64-1.47)	0.49 (0.27-0.87)	0.42 (0.22-0.80)

[a]Bioavailable testosterone calculated from the equation "(testosterone/SHBG) X 10"
[b]Number may vary due to missing values
[c]Women in luteal phase are excluded
[d]Bioavailable testosterone divided by the standard deviation; Nasal carriage all women SD=0.19; Nasal carriage premenopausal SD=0.34; Nasal carriage postmenopausal SD=0.09; Persistent carriage all women SD=0.19; Persistent carriage premenopausal SD=0.35; Persistent carriage postmenopausal SD=0.09
[e]Adjusted for BMI, age and HbA1c
[f]Adjusted for BMI, age, HbA1c and smoking
[g]Adjusted for BMI, age, HbA1c, smoking and alcohol use
[h]Adjusted for BMI, age, HbA1c, smoking alcohol use and hospital admission
[i]Adjusted for BMI, age, HbA1c, alcohol use, hospital admission and vitamin D
