# Supplementary material for: Circulating sex-steroids and Staphylococcus aureus nasal carriage in a general female population
Source: Eur J Endocrinol. 2020 Dec 16;184(2):337–46. doi: 10.1530/EJE-20-0877 (PMC7849480; doi:10.1530/EJE-20-0877)
Supplement: Supplementary Table 5: Associations between bioavailable testosterone and Staphylococcus aureus nasal carrier and persistent carrier state by Morris et ala. Odds ratios (ORs) and 95% confidence intervals (95% CIs) from logistic regression analysis. The Tromsø Study 6 [file supplementary_table_5.pdf]

Supplementary Table 5: Associations between bioavailable testosterone and *Staphylococcus aureus* nasal carrier and persistent carrier state by Morris et al[a]. Odds ratios (ORs) and 95% confidence intervals (95% CIs) from logistic regression analysis. The Tromsø Study 6

	Nasal carrier			Persistent nasal carrier		
	**All women** (n=561)	**Pre-menopausal** (n=148)	**Post-menopausal** (n=414)	**All women** (n=548)	**Pre-menopausal** (n=135)	**Post-menopausal** (n=414)
**Bioavailable testosterone, pr SD[a]**	0.58 (0.37-0.92)	0.27 (0.05-1.43)	0.78 (0.59-1.02)	0.55 (0.33-0.91)	0.33 (0.05-2.20)	0.73 (0.54-0.98)
**BMI, kg/m^2**	1.03 (1.00-1.07)	1.09 (1.02-1.16)	1.01 (0.97-1.06)	1.04 (1.00-1.08)	1.10 (1.02-1.18)	1.01 (0.97-1.06)
**Age, year**	0.99 (0.98-1.01)	0.98 (0.90-1.06)	0.99 (0.97-1.02)	1.00 (0.98-1.01)	0.98 (0.90-1.08)	1.00 (0.97-1.03)

[a]ln BioT = -0.266 + (0.955 x lnTT) – (0.228 x lnSHBG)

[b]Bioavailable testosterone divided by the standard deviation; Nasal carriage all women SD=0.23; Nasal carriage premenopausal SD=0.40; Nasal carriage postmenopausal SD=0.12; Persistent carriage all women SD=0.23; Persistent carriage premenopausal SD=0.43; Persistent carriage postmenopausal SD=0.12
